# Comparative long-term trend analysis of daily weather conditions with daily pollen concentrations in Brussels, Belgium

**DOI:** 10.1007/s00484-017-1457-3

**Published:** 2017-10-24

**Authors:** Nicolas Bruffaerts, Tom De Smedt, Andy Delcloo, Koen Simons, Lucie Hoebeke, Caroline Verstraeten, An Van Nieuwenhuyse, Ann Packeu, Marijke Hendrickx

**Affiliations:** 1 Service Mycology & Aerobiology, Scientific Institute of Public Health, Rue Wytsmanstraat 14, 1050 Brussels, Belgium; 2 Service Health & Environment, Scientific Institute of Public Health, Brussels, Belgium; 3 Present Address: P-95, Heverlee, Belgium; 4 Ozone, UV and Aerosols Group, Royal Meteorological Institute of Belgium, Brussels, Belgium; 5 Present Address: Centre for Epidemiology and Biostatistics, Melbourne School of Population and Global Health, University of Melbourne, Melbourne, VIC Australia

**Keywords:** Pollen, Climate change, Meteorological factors, Allergy

## Abstract

A clear rise in seasonal and annual temperatures, a gradual increase of total radiation, and a relative trend of change in seasonal precipitation have been observed for the last four decades in Brussels (Belgium). These local modifications may have a direct and indirect public health impact by altering the timing and intensity of allergenic pollen seasons. In this study, we assessed the statistical correlations (Spearman’s test) between pollen concentration and meteorological conditions by using long-term daily datasets of 11 pollen types (8 trees and 3 herbaceous plants) and 10 meteorological parameters observed in Brussels between 1982 and 2015. Furthermore, we analyzed the rate of change in the annual cycle of the same selected pollen types by the Mann-Kendall test. We revealed an overall trend of increase in daily airborne tree pollen (except for the European beech tree) and an overall trend of decrease in daily airborne pollen from herbaceous plants (except for Urticaceae). These results revealed an earlier onset of the flowering period for birch, oak, ash, plane, grasses, and Urticaceae. Finally, the rates of change in pollen annual cycles were shown to be associated with the rates of change in the annual cycles of several meteorological parameters such as temperature, radiation, humidity, and rainfall.

**Electronic supplementary material:**

The online version of this article (10.1007/s00484-017-1457-3) contains supplementary material, which is available to authorized users.

## Introduction

Prevalence of chronic respiratory diseases, including allergic rhinitis and asthma, has been estimated to reach up to 40% in European countries (D’Amato et al. 2007). This problem is all the more complex as multiple factors need to be considered such as the evolution of our way of life and various environmental changes. For instance, climate factors, together with air pollution, lead to a wide range of impacts on vegetation, altering specific phenological phases and consequently the production and release of pollen in the air (D’Amato et al. 2016). It is expected that climate change may induce an increase in airborne pollen, an increase of the allergen potency of pollen grains, and variations in the pollen season timing, with an earlier onset and a longer duration. Numerous studies already addressed the tight relations between aerobiology and biometeorology (Beggs et al. 2017). They demonstrate the clear short- and long-term influence of meteorological parameters (temperature, radiation, relative humidity, rainfall, wind speed) on airborne pollen season. However, these consequences may significantly vary from one region to another, depending on the selected taxon and on the clime (Donders et al. 2014; Emberlin et al. 2002; Galán et al. 2005). Indeed, the complexity of interactions and overlapping actions between meteorological parameters, the plant physiognomy and phenology, the land use, and other environmental factors presents a serious challenge for researchers to develop robust and precise pollen forecast models (Novara et al. 2016; Sofiev et al. 2015). Pollen monitoring thus plays a crucial role to improve these models by feeding them with historical data.

In Belgium, the pollen and fungal spore concentrations in the air have been continuously monitored by the Belgian Aerobiological Surveillance Network since 1982 in Brussels. In addition to the precious “real-time” information it represents for allergy sufferers and medical practitioners, the Belgian pollen database allows to address the long-term evolution of the timing and intensity of the pollen seasons (Detandt and Nolard 2000; Spieksma et al. 2003; Ziello et al. 2012). Based on the most recent analysis of annual datasets collected from 1982 to 2015 (unpublished data), an increase of airborne pollen for alder, hazel, birch, ash, and plane trees was pointed out, whereas airborne pollen from herbaceous plants (grasses, mugwort) was shown to decrease. These results actually improve upon the conclusions made by the previous Belgian studies based on the annual pollen index recorded between 1982 and 2001 and in which cyclic interannual variability in tree airborne pollen was observed, while no particular trend could be demonstrated (Detandt and Nolard 2000; Spieksma et al. 2003). This can probably be attributed to a lack of statistical power. Primarily, these older studies are based on shorter time series. In addition, the contrast in meteorological observations is larger in the more recent study: the last 10 years appeared to be warmer than between 1982 and 2001, with the highest annual mean temperatures ever recorded since 1833 (11.3 °C in 2015, 11.9 °C in 2014, 11.6 °C in 2011, and 11.5 °C in 2007) (Belgian Federal Public Service 2017). These favorable conditions would therefore tend to reduce the differences in the total pollen concentrations that are observed between the interannual cycles of anemophilous trees in Belgium (Detandt and Nolard 2000). Indeed, other annual-scaled studies show that airborne pollen strongly depends on specific combination of meteorological variables and of physiology and phenology of plants (Hicks 2001; Nielsen et al. 2010).

The first attempt to associate the airborne release of pollen with meteorological conditions in Belgium was the study reported by Emberlin et al. (2002), in which an overall trend toward earlier start dates for the birch pollen season was observed along with increasing warmth. Nevertheless, this study was limited to one taxon, and the statistical analysis was performed with only one pollen season parameter (start date) vs. one meteorological parameter (mean temperature). The seasonal temperatures (minimal, maximal, and mean) are not the only parameters that have been observed to rise in Belgium (Vandiepenbeeck et al. 2015). This evolution has been coupled with a gradual increase of total radiation. In the Brussels Capital Region, this warming was attributed to regional impact of climate change but also to the intensifying urban heat island effects (Hamdi et al. 2009). Moreover, the combined effects of climate change and urbanization on the precipitation scenario were estimated to exacerbate surface runoff, water high flow, and flood event frequency in a case study undertaken in the Brussels Capital Region (Hamdi et al. 2011). Indeed, precipitation and extreme rainfall events in winter and spring have been increasing, and the five highest frequency values of the last hundred years have been measured in Brussels during the last 13 years. The significance of this increasing trend remains to be demonstrated over the long term. Notable changes of the seasonal precipitation levels are anticipated in the future, increasing in winter and spring and decreasing in summer (Belgian Federal Public Service 2017).

Given this significant change in the local weather conditions in Brussels, this study intends to estimate the statistical correlations between several meteorological parameters and airborne pollen from 11 taxa (8 tree taxa and 3 herbaceous plant taxa), as well as the association between changes in annual patterns of these variables and concentrations. The purpose is to give a more comprehensive view of the potential regional impact of climate change on the airborne pollen season in Belgium, which may consequently affect public health by causing allergies.

## Materials and methods

### Study area

**Fig. 1:**
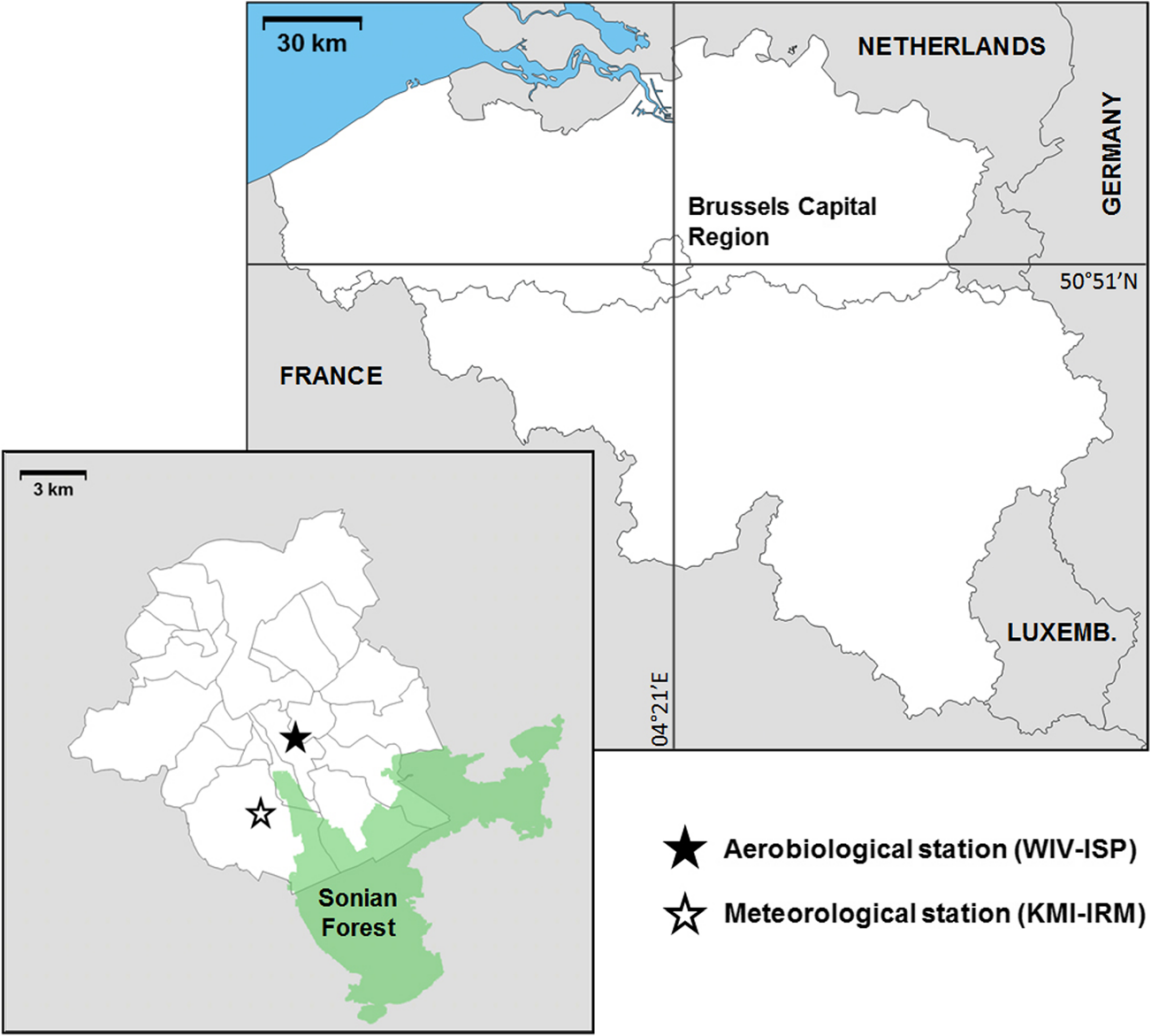
The aerobiological and meteorological stations within the Brussels Capital Region (lower panel), which are located at 50° 51′ N, 04° 21′ E in Belgium (upper panel). WIV-ISP: Scientific Institute of Public Health, KMI-IRM: Royal Meteorological Institute

The aerobiological station and the meteorological station used for this study are localized in the Brussels Capital Region, at 3.2 km from each other (Fig. 1). Satellite mapping studies estimate the visible vegetation cover of approximately 54% of the region surface (Van de Voorde et al. 2010). Brussels is therefore considered as one of the greenest European capital cities. Regional streets are mainly tree-lined with beech, horse chestnut, maple, and plane. Notably, the Sonian Forest is localized at only 4 km distance SSE from the aerobiological station. This ancient peri-urban forest (4400 ha) is dominantly composed of beech trees (*Fagus sylvatica*) (69% of the tree composition), but it also comprises other tree taxa such as oak (*Quercus* spp.) (15%), ash (*Fraxinus excelsior*), alder (*Alnus* spp.), hornbeam (*Carpinus betulus*), and birch (*Betula* spp.) (< 2%) (Van der Perre et al. 2014). The area at an approximate 10-km radius distance around the aerobiological station includes the entire Brussels Capital Region’s territory. Beyond this distance, the region is enclosed by the Flemish and Walloon Brabant provinces (from 10 to 50 km around the station), which are characterized by larger green areas such as forest, grassland, and agricultural land.

Regarding the temperate climate zone in Brussels, the mean annual temperature between 1982 and 2015 was 10.5 °C, with a tendency to increase (up to 11.9 °C in 2014). The coldest month is January with a mean temperature of 3.3 °C, and the hottest mean temperature is recorded in July with 18.4 °C. The annual average total precipitation (852.4 mm) is relatively high as compared to other European capitals (Belgian Federal Public Service 2017; Vandiepenbeeck et al. 2015).

### Pollen data

Pollen monitoring in Brussels has been continuously performed since 1982 by the Belgian Aerobiological Surveillance Network (http://www.airallergy.be). Pollen grains are sampled by the use of a 7-day Hirst-type volumetric spore trap (Hirst 1952), placed on the top of the roof of the Scientific Institute of Public Health (WIV-ISP, site Elsene-Ixelles), at 16 m height above the ground level. The methodological guidelines requirements by the European Aerobiology Society (EAS) were followed to the best ability (Galán et al. 2014), apart from the average daily pollen concentration (pollen/m^3^) which was calculated from the counting of 8% slide surface (two longitudinal lines reading). Data were recorded from January 1st to September 30th between 1982 and 2015, with missing values never exceeding 2 weeks for any taxon. The following 11 taxa were selected: alder (*Alnus* spp.), hazel (*Corylus avellana*), birch (*Betula* spp.), hornbeam (*Carpinus betulus*), beech (*Fagus sylvatica*), oak (*Quercus* spp.), ash (*Fraxinus excelsior*), plane (*Platanus* spp.), grasses (Poaceae), mugwort (*Artemisia* spp.), and Urticaceae (this includes pollen from nettles (*Urtica* spp.) and pellitories (*Parietaria* spp.)). Date of the start/end of the pollen season is defined as the day when the cumulative pollen concentration reaches 5%/95% of the annual pollen index.

### Meteorological data

Monitoring of the meteorological parameters was performed at the Uccle Observatory (in place since 1886) by the Belgian Royal Meteorological Institute (KMI-IRM). Average daily data were strictly calculated for mean/max/min temperature (°C), total radiation (W/m^2^), mean/max/min relative humidity (%), rainfall (mm), wind speed (m/s), and wind calm frequency (%).

### Statistical analysis

Correlation between daily pollen and meteorological datasets was evaluated by performing the nonparametric Spearman correlation test. For this analysis, data were restricted to the airborne pollen seasons. Nonparametric statistics were chosen because normal distribution of this type of data was not expected. This was also assessed using a Shapiro-Wilk test of normality and through visual inspection of the data.

Rate of change in annual cycles for pollen and meteorological datasets was evaluated by calculating the Sen slope value for each day of the season (Makra et al. 2011). Subsequently, the rate of change in the annual cycle was smoothed with the local regression method (LOESS) (García-Mozo et al. 2014), with span equal to 0.1. The *area under the curve* (AUC) was calculated by the trapezoidal method. Association measure between the rates of change in the annual cycles of pollen concentrations and meteorological parameters was performed by the Mann-Kendall test.

Statistical analyses were performed by using the R programming environment 3.2.2 (Foundation for Statistical Computing, Vienna, Austria), and plots with calculated AUC were generated with GraphPad Prism software 5.0 (San Diego, CA, USA).

## Results

### Correlations between pollen concentrations and meteorological parameters

In a first step, we plotted the daily pollen concentrations of 11 selected taxa vs. 10 meteorological parameters for each available data (Figure Appendices 1–3). The Spearman correlation reflects the main influence of the weather conditions on the airborne pollen concentrations on the dispersal day (Table 1). As expected, we observed more elevated pollen concentrations during warm and dry days. In contrast, wind-related parameters presented low correlation values, i.e., under 0.09, for almost all pollen taxa. Birch pollen concentrations were negatively correlated with temperature variables (*p* < 0.001), whereas no significant correlation could be demonstrated for alder pollen. The radiation levels, usually associated with temperature, show also significant positive correlation with pollen concentrations (with the exception of birch pollen). The highest significant dependence to the weather conditions on the dispersal day was measured for the selected herbaceous plants, namely grasses, mugwort, and the Urticaceae. Moreover, for these plants which flower in summer, absolute correlation values with both temperature and radiation variables were largely above 0.09. In contrast, correlation was stronger with temperature variables than with radiation, in regard to the selected tree taxa flowering in winter and early spring.

**Table 1:** Correlation between daily pollen concentrations and daily meteorological parameters. Absolute correlation values above 0.09 are indicated in bold

Pollen taxon	Family	Temperature			Radiation	Relative humidity			Rainfall	Wind speed	Wind calm frequency
		Mean	Max	Min		Mean	Max	Min			
Alder	*Betulaceae*	0.004	0.049	− 0.029	0.011	− 0.126***	− 0.091***	− 0.112***	− 0.095***	0.057*	− 0.074**
Hazel	*Betulaceae*	0.169***	0.182***	0.157***	− 0.022	− 0.151***	− 0.178***	− 0.106***	− 0.103***	0.184***	− 0.154***
Birch	*Betulaceae*	− 0.209***	− 0.131***	− 0.278***	− 0.067**	− 0.198***	− 0.173***	− 0.202***	− 0.141***	0.066	− 0.031
Hornbeam	*Betulaceae*	0.264***	0.255***	0.228***	0.009	− 0.171***	− 0.163***	− 0.141***	− 0.053	0.105*	− 0.055
Beech	*Fagaceae*	0.153**	0.174***	0.098*	0.018	− 0.099*	− 0.069	− 0.099*	− 0.058	− 0.085	0.147**
Oak	*Fagaceae*	0.202***	0.237***	0.106**	0.148***	− 0.268***	− 0.210***	− 0.275***	− 0.168***	− 0.012	0.036
Ash	*Oleaceae*	0.153***	0.170***	0.086*	0.067*	− 0.178***	− 0.146***	− 0.179***	− 0.087**	0.005	0.005
Plane	*Platanaceae*	0.150***	0.189***	0.063	0.093**	− 0.234***	− 0.197***	− 0.261***	− 0.119***	− 0.066	0.168***
Grass	*Poaceae*	0.363***	0.366***	0.274***	0.449***	− 0.271***	− 0.207***	− 0.216***	− 0.149***	− 0.055**	− 0.008
*Urticaceae*	*Urticaceae*	0.574***	0.547***	0.470***	0.355***	− 0.269***	− 0.196***	− 0.235***	− 0.259***	− 0.172***	0.150***
Mugwort	*Asteraceae*	0.249***	0.251***	0.180***	0.200***	− 0.136***	− 0.044	− 0.139***	− 0.168***	− 0.089*	0.025

*p < 0.05; **p < 0.01; ***p < 0.001

### Rate of change in the annual cycle of pollen concentrations and meteorological parameters

**Fig. 2:**
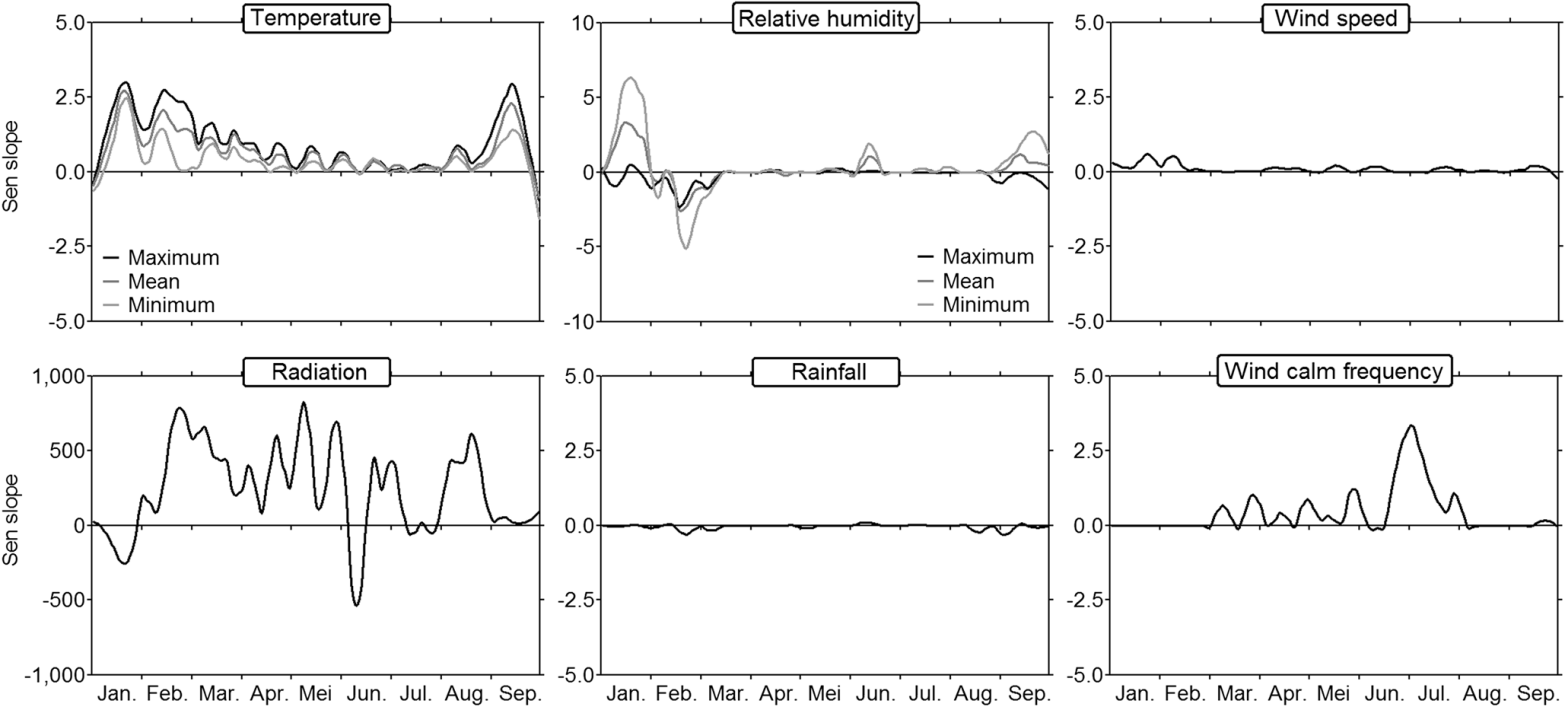
Rate of change in the annual cycles of meteorological parameters between 1982 and 2015. Trend values are calculated from the Sen slope for each day of the season and represented by a curve after LOESS smoothing (span value = 0.1)

Long-term trends were estimated individually for each day of the season, as initially described by Makra et al. (2011). The rate of change in the annual cycle of the selected meteorological parameters (Fig. 2) presents highly variable patterns, even after this smoothing process. Temperature variables (mean, maximum, and minimum) tend to have increased these last decades, mostly on January, February, March, and September. Wide variations can be clearly observed between the minimum temperature trend curve and the mean and maximum temperature curves. Radiation trend pattern appears to fluctuate, with negative daily trend values in January and early June. Relative humidity variables are the most divergent between the mean, maximum, and minimum variables. Trend values in some time intervals even appear to be opposite (January, February, and September). Notably, the rainfall trend curve seems to converge rather with the trend curve of maximum humidity, as shown by negative values on February and August. Finally, regarding wind variables, while the wind speed has been tending to increase in January and February, wind calm frequency has been overall increasing from March to July in the last 34 years.

**Fig. 3:**
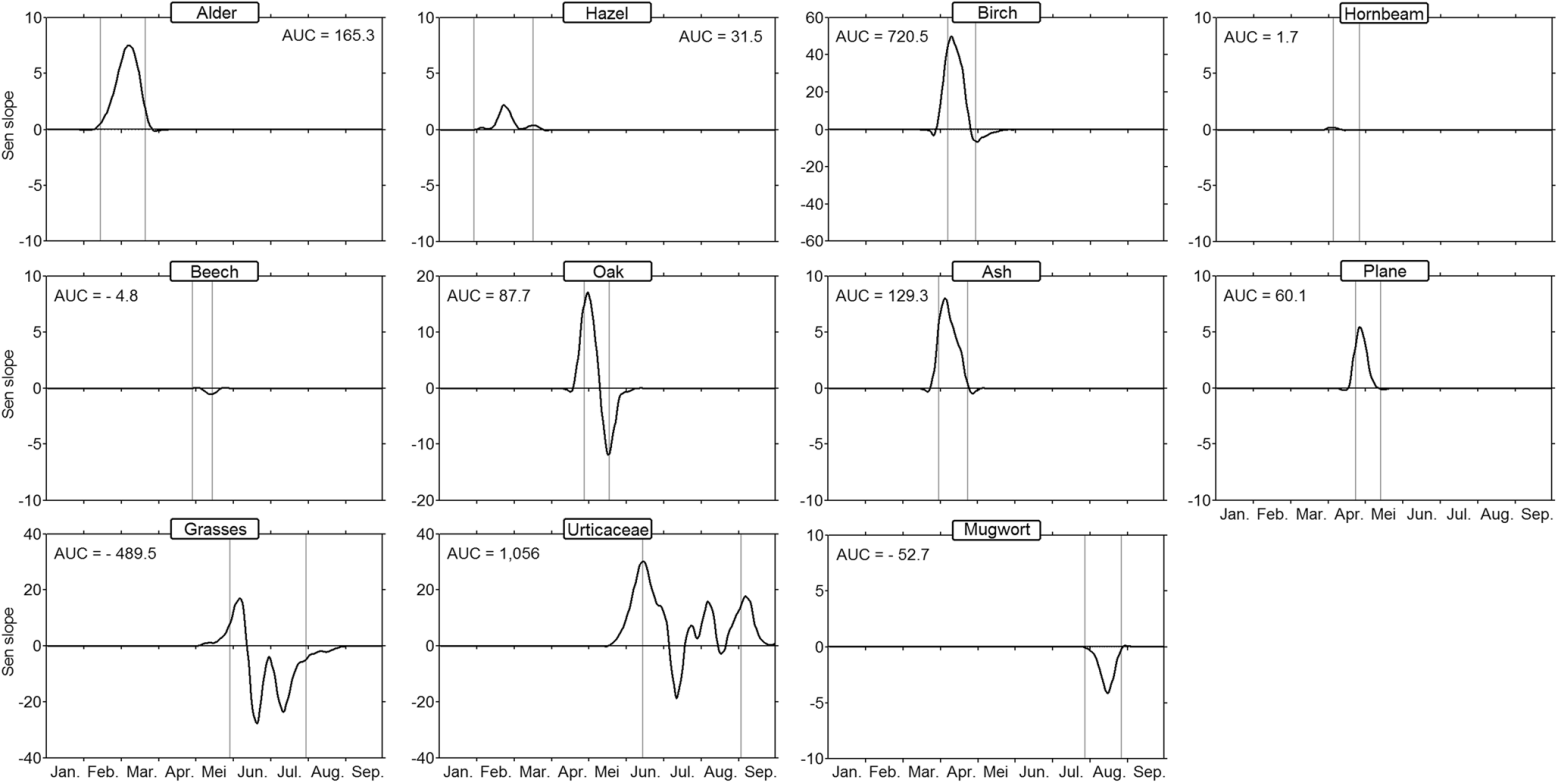
Rate of change in the annual cycles of pollen concentrations between 1982 and 2015. Trend values are calculated from the Sen slope for each day of the season and represented by a curve after LOESS smoothing (span value = 0.1). Average season start/end (calculated from the cumulative 5%/95% of the annual pollen index) is represented with vertical gray lines. AUC: area under the curve

The rate of change in annual cycle for pollen concentrations was depicted within the time intervals defined by the average start and end of each specific pollen season (Fig. 3). The pollen concentration trends of alder and hazel have been increasing approximately in the middle of their respective flowering period (February), whereas positive trends were observed at the start of the birch, hornbeam, ash, and plane flowering periods. This suggests the tendency for these last trees to start their pollination period earlier over the years. Interestingly, oak pollen concentrations have been tending to increase at the mean of the pollen season start and to decrease at the mean end. This highlights a remarkable time shift of the oak pollen season, which gradually tends to overlap with the birch pollen curve. For beech pollen, we observed a decreasing trend in airborne concentrations in May. Regarding herbaceous plants, the generated trend curves for Poaceae and Urticaceae revealed highly variable patterns, probably due to the number of different species composing these families. The grass pollen season appears to start more intensely while tending to be milder in June and July. The Urticaceae pollen season tends to form two distinct waves, one in May–June and another in late summer. Finally for mugwort, lower pollen concentrations have clearly been detected during their pollen season in August.

In contrast to the methodology reported by Makra et al. (2011), we did not average the values of daily slopes of linear trends to give rates of change of annual airborne pollen. Instead, we calculated the total AUC from the cycles of daily slopes. This numerical value of a definite integral allows to reveal an alternative index of the overall change of the annual airborne pollen. It provides an interesting indication because the time intervals with positive and negative trends are highly variable within the year. It is worth mentioning, however, that it is difficult to compare the absolute AUC values between each pollen taxa because they are directly dependent to the specific abundance of pollen produced by each plant. As shown in Fig. 3, the highest positive AUC values were observed for the pollen emitted by birch (AUC = 720.5) and the Urticaceae (AUC = 1056). To a lesser extent, positive AUC values were also observed for tree pollen from alder, hazel, ash, oak, and plane. In contrast, a decrease of the annual airborne pollen was shown for pollen produced by herbaceous plants, such as mugwort and more particularly grasses (AUC = − 489.5).

### Associations between the rates of change in the annual cycles

Association measures were analyzed by correlating each rate of change in the annual pollen cycles with each rate of change in the annual cycle of the meteorological variables (Table 2). The temperature trends were positively associated with the pollen concentration trends observed for all taxa (*p* < 0.001), except for plane and the Urticaceae. Indeed, these two last taxa presented significantly negative association values (*p* < 0.05 to *p* < 0.001). Daily trends of radiation over the years were also positively associated with almost all pollen taxa, whereas beech and the Urticaceae showed a significant negative association (*p* < 0.001). Inversely, trends for relative humidity variables and rainfall were generally negatively associated with the trends of pollen concentrations, again except for plane, beech, and the Urticaceae. For a given pollen taxon, high differences are also observed between the association value for the maximum relative humidity and the association value for the minimum or mean relative humidity. In some cases, association values with maximum relative humidity are lower (birch, hornbeam) or higher (alder, hazel) than with the minimum or mean variable. For beech, plane, and mugwort pollen, this association is totally inverted, probably due to the high variability in the daily trends shown for these humidity parameters. The relative humidity cycles are even opposite in some time intervals, as shown in Fig. 3.

**Table 2:** Association measure between the rates of change in the annual cycles for pollen concentrations and the rate of change in the annual cycles for meteorological parameters. Absolute correlation values above 0.2 are indicated in bold

Pollen taxon	Family	Temperature			Radiation	Relative humidity			Rainfall	Wind speed	Wind calm frequency
		Mean	Max	Min		Mean	Max	Min			
Alder	*Betulaceae*	0.53***	0.52***	0.51***	0.18***	− 0.29***	− 0.53***	− 0.30***	− 0.52***	− 0.09*	− 0.40***
Hazel	*Betulaceae*	0.67***	0.66***	0.65***	0.01	− 0.12**	− 0.69***	− 0.13***	− 0.60***	0.02	− 0.51***
Birch	*Betulaceae*	0.19***	0.18***	0.13**	0.59***	− 0.39***	− 0.01	− 0.38***	− 0.18***	− 0.11**	− 0.18***
Hornbeam	*Betulaceae*	0.23***	0.22***	0.17***	0.60***	− 0.41***	− 0.05	− 0.40***	− 0.21***	− 0.10*	− 0.22***
Beech	*Fagaceae*	0.39***	0.41***	0.41***	− 0.42***	0.29***	− 0.59***	0.28***	− 0.46***	− 0.02	− 0.30***
Oak	*Fagaceae*	0.20***	0.19***	0.14***	0.51***	− 0.32***	− 0.02	− 0.30***	− 0.21***	− 0.12**	− 0.22***
Ash	*Oleaceae*	0.20***	0.19***	0.13**	0.61***	− 0.40***	− 0.02	− 0.40***	− 0.18***	− 0.10*	− 0.19***
Plane	*Platanaceae*	− 0.09*	− 0.10*	− 0.15***	0.59***	− 0.39***	0.27***	− 0.38***	0.08	− 0.06	0.07
Grass	*Poaceae*	0.26***	0.26***	0.19***	0.32***	− 0.10*	− 0.11**	− 0.09*	− 0.25***	− 0.08	− 0.34***
*Urticaceae*	*Urticaceae*	− 0.66***	− 0.65***	− 0.60***	− 0.23***	0.13**	0.48***	0.13**	0.51***	− 0.15***	0.60***
Mugwort	*Asteraceae*	0.02	0.00	− 0.03	0.34***	− 0.14**	0.19***	− 0.15***	0.30***	0.37***	0.02

*p < 0.05; **p < 0.01; ***p < 0.001

With regard to the wind-related parameters, wind speed appears to be associated with almost none of the selected pollen taxa, as shown by nonsignificant and/or low absolute correlation values. Wind calm frequency is in contrast negatively associated with all the selected pollen taxa, with the exception of plane, mugwort (no significant association), and the Urticaceae (significantly positive association).

The strength of the associations with meteorological variables may also be analyzed by clustering groups of tree taxa that share nearly the same flowering period of the year. For instance, alder and hazel, which flower from end of January to early March in Belgium, showed among tree taxa the highest significantly positive association values with temperature variables and the highest negative association values with rainfall and wind calm frequency. The specific profile of the daily trends of these two trees seems to be influenced by the long-term warming observed in this period (Fig. 2). Regarding the trend of increasing radiation observed from mid-February, the association value did not appear to be as high as it was for temperature variables. This is probably due to the fact that the long-term increase of radiation occurred too late after the average period of the peak concentration for alder and hazel, which notably represents 20% of their annual pollen index. In contrast, association values were significantly higher between the daily radiation trends, which were higher on spring, and the daily pollen trends for all the other trees (with the exception of beech).

## Discussion

The meteorological study of climate change has been undertaken for many years in Belgium, as it is reported by the Royal Meteorological Institute in its most recent Climate Surveillance Report (Vandiepenbeeck et al. 2015). Our study aims to highlight the consequent impact on the airborne pollen concentrations, which may be considered on one hand as a downstream indicator of plant fitness and population dynamics and on the other hand as a risk indicator of clinical allergy. Indeed, these climate-induced modifications may critically increase the risk of exposure to aeroallergens (D’Amato et al. 2016).

We have shown that pollen concentration trends were associated with the overall increasing trend of temperature and radiation and inversely associated with the fluctuating trends of relative humidity and rainfall. We have also confirmed, but for the first time on a daily data-based analysis, the increasing trend of pollen concentrations for alder, hazel, birch, ash, and plane, as well as the decreasing trend for grasses and mugwort. These results are consistent with our previous work (unpublished data), in which the long-term trend was linearly analyzed from total annual pollen datasets. Moreover, pollen concentrations of several taxa were shown to have increased during the start of each specific season. This information is of particular importance since this period is described as more critical for allergic sufferers, due to a “priming effect” of allergy reactions (Pfaar et al. 2017). As well, earlier exposure often involves the extension of the season period and sometimes a shift of the period (as illustrated here for oak trees). It is tempting to suggest that these timing modifications might be attributed to climate-related or other environmental (pollution, biogeochemical) factors rather than land cover-related factors because a change in plant distribution would reasonably induce an increase or a decrease in pollen concentration trends in the overall season period (García-Mozo et al. 2016).

However, the association of the daily trends for beech pollen with meteorological variables is rather counterintuitive, i.e., negative with radiation, positive with mean and minimum relative humidity, and much stronger with temperature and rainfall than it is for other tree taxa flowering at the same period. This may partly be explained by the fact that the estimates for the effects of meteorological parameters are biased by confounding with other nonclimatic factors such as the local evolution of plant distribution (Galán et al. 2016; García-Mozo et al. 2016). However, this is unlikely given that the reported land use data in Brussels indicates a relatively stable forest total surface (+ 2%), mainly allocated to the beech-composed Sonian Forest, from 1982 to 2015 (Belgian Federal Statistics 2017). But this data might also be masked by a gradual change in the tree composition of the forest and should be further investigated with a long-term tree-specific inventory, or by studying the behavior of these trees in their ecological niche. In this respect, the monitoring of tree pollen in the air may be a precious indicator of vitality, growth, and composition of this forest, as beech trees (representing 69% of the tree composition) would be highly susceptible to modifications of some climate-related parameters such as temperature and precipitation levels (Campioli et al. 2012; Van der Perre et al. 2014).

In regard to the pollen produced by the four species from the Urticaceae family present in Belgium (*Urtica dioica*, *Urtica urens*, *Parietaria officinalis*, and *Parietaria judaica*), descriptive analysis of the associations with meteorological variables appears to be difficult because of the complexity of the trend cycle of these herbaceous plants. Associations were shown to be overall inverted as compared to other taxa. In contrast to other herbaceous plants and undershrubs, Urticaceae are known to display highly specific ecological behavior, which is reflected by a distinct adaptation to climate and environmental changes (Galán et al. 2016). Potential climatic and nonclimatic forces are probably multiple and may interfere at distinct time intervals during this very long flowering period. Indeed, even if availability of open land surface in the Brussels Capital Region has been drastically reduced for three decades (− 56% agricultural land, − 45% meadow, − 40% wasteland (Belgian Federal Statistics 2017)), soil eutrophication has been highly favoring proliferation of nettles, whereas adaptation of pellitories has also been observed locally alongside the banks of rivers and canals in several Belgian urban areas (Martin 2004).

Airborne pollen directly depends on sequential phenological steps before and during flowering. This represents as many time points on which climate factors may have an impact (García-Mozo et al. 2015). Interestingly, budburst has significantly increased as a consequence of global warming. Modeling and field studies estimate that both winter and spring warming may significantly advance budburst date of beech, birch, and oak trees (Fu et al. 2012; Kuster et al. 2014). These results are quite surprising since chilling units are known to be important for budburst. The release from winter dormancy is environmentally mediated by species-specific responses to temperature and photoperiod. The “chilling and heating” mechanism is similarly described for the flowering phase and pollen release, especially for alder and hazel in early year, and so has been used for the development of pollen forecast models (Novara et al. 2016). However, our study does not take into account the chill accumulation during the previous winter period. The analysis of lagged time series for pollen concentration and meteorological data would though bring more understanding on this phenomenon (Guilbert et al. 2016).

Furthermore, our results revealed some discrepancies between the association values (Table 2) and the strict correlation values (Table 1) with the meteorological parameters. For instance, low absolute association values and very high correlation values were observed for pollen concentrations of the herbaceous plants in regard to relative humidity. Yet, the herbaceous plants have consistently been shown to present a higher climate sensitivity, especially water sensitivity, as compared to arboreal taxa (Galán et al. 2016; Matyasovszky et al. 2015). This illustrates the differential influence of meteorological variables that may act on the pollen emission on one hand and on the pollen dispersal on the other. Indeed, the correlation values rather describe that the dry conditions are simply favoring the pollen transport in the air, whatever the timing of the season. In contrast, the association value between the trends at a particular day involves the temporality of the season and therefore the phenological stages. This may rather reflect the overall effects of a given meteorological factor on the long-term evolution of the pollen concentration, which act on both emission and dispersal levels. For example, dry conditions are optimal for pollen dispersal, but the lack of water during or a few months prior the phenological flowering stage also induces a reduction in the annual pollen index, depending on the species (Galán et al. 2016). These overlapping effects therefore tend to reduce the long-term trend association values shown in our study. Nonetheless, herbaceous plants usually display more immediate response to weather conditions than trees, as illustrated by our correlation results.

## Conclusions

In addition, to help in the evaluation of the pollinosis risk, daily airborne pollen concentrations represent an interesting indicator to measure the long-term effects of environmental changes on vegetation. This study revealed an overall trend of increase in daily airborne tree pollen (except for beech) and an overall trend of decrease in daily airborne pollen from herbaceous plants (except for the Urticaceae) for 34 years in Brussels. These trends were shown to be correlated with the gradual change of meteorological parameters such as temperature, radiation, humidity, and rainfall. However, this taxon-specific sensitivity to climate change is potentially confounded with many other interactive factors such as air pollution, biogeochemical factors, and land use. A global approach should therefore be undertaken in order to better understand the dynamics of these factors and to evaluate their combined and weighted impact on the evolution of pollen concentrations in the air and on the consequent risk of developing allergy disease.

## Electronic supplementary material

ESM 1 Scatter plots of daily pollen concentrations for 4 Betulaceae tree taxa (A: alder, B: hazel, C: birch, D: hornbeam) vs. daily measures of 10 meteorological parameters. Datasets include measures between January 1^st^ and September 30^th^, from 1982 to 2015. Trend was represented by linear regression.

ESM 2 Scatter plots of daily pollen concentrations for 4 tree taxa (A: beech, B: oak, C: ash, D: plane) vs. daily measures of 10 meteorological parameters. Datasets include measures between January 1^st^ and September 30^th^, from 1982 to 2015. Trend was represented by linear regression.

ESM 3 Scatter plots of daily pollen concentrations for 3 herbaceous plant taxa (A: grasses, B: Urticaceae, C: mugwort) vs. daily measures of 10 meteorological parameters. Datasets include measures between January 1^st^ and September 30^th^ , from 1982 to 2015. Trend was represented by linear regression.

## Abbreviations

AUC: Area under the curve
LOESS: Local regression method
WIV-ISP: Belgian Scientific Institute of Public Health
KMI-IRM: Belgian Royal Meteorological Institute
