# Supplementary figures and images for: Comparative long-term trend analysis of daily weather conditions with daily pollen concentrations in Brussels, Belgium
Source: Int J Biometeorol. 2017 Oct 24;62(3):483–91. doi: 10.1007/s00484-017-1457-3 (PMC5854748; doi:10.1007/s00484-017-1457-3)

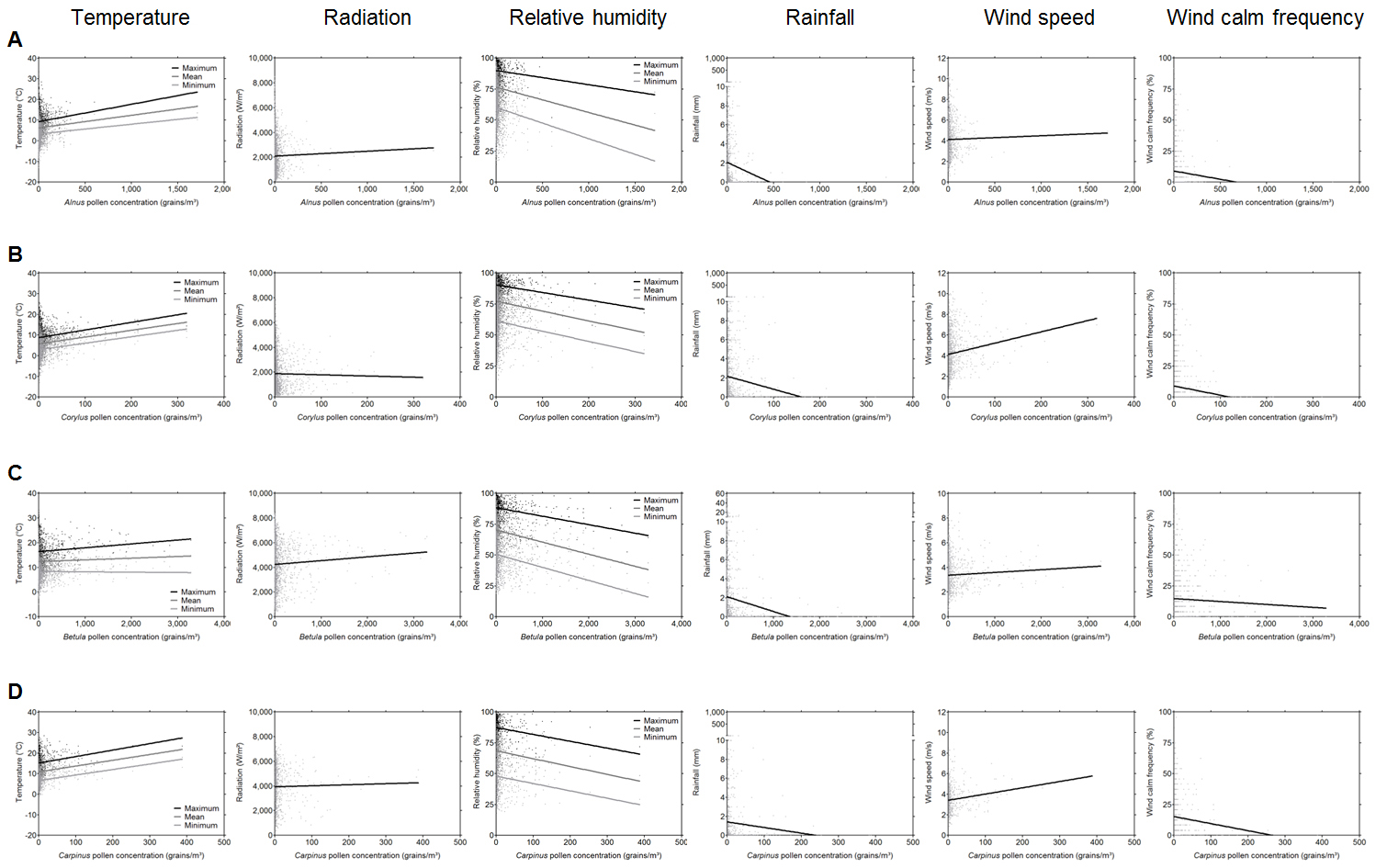

Supplement: Supplementary file 1 — Scatter plots of daily pollen concentrations for 4 Betulaceae tree taxa (A: alder, B: hazel, C: birch, D: hornbeam) vs. daily measures of 10 meteorological parameters. Datasets include measures between January 1st and September 30th, from 1982 to 2015. Trend was represented by linear regression. [file 484_2017_1457_MOESM1_ESM.jpg]

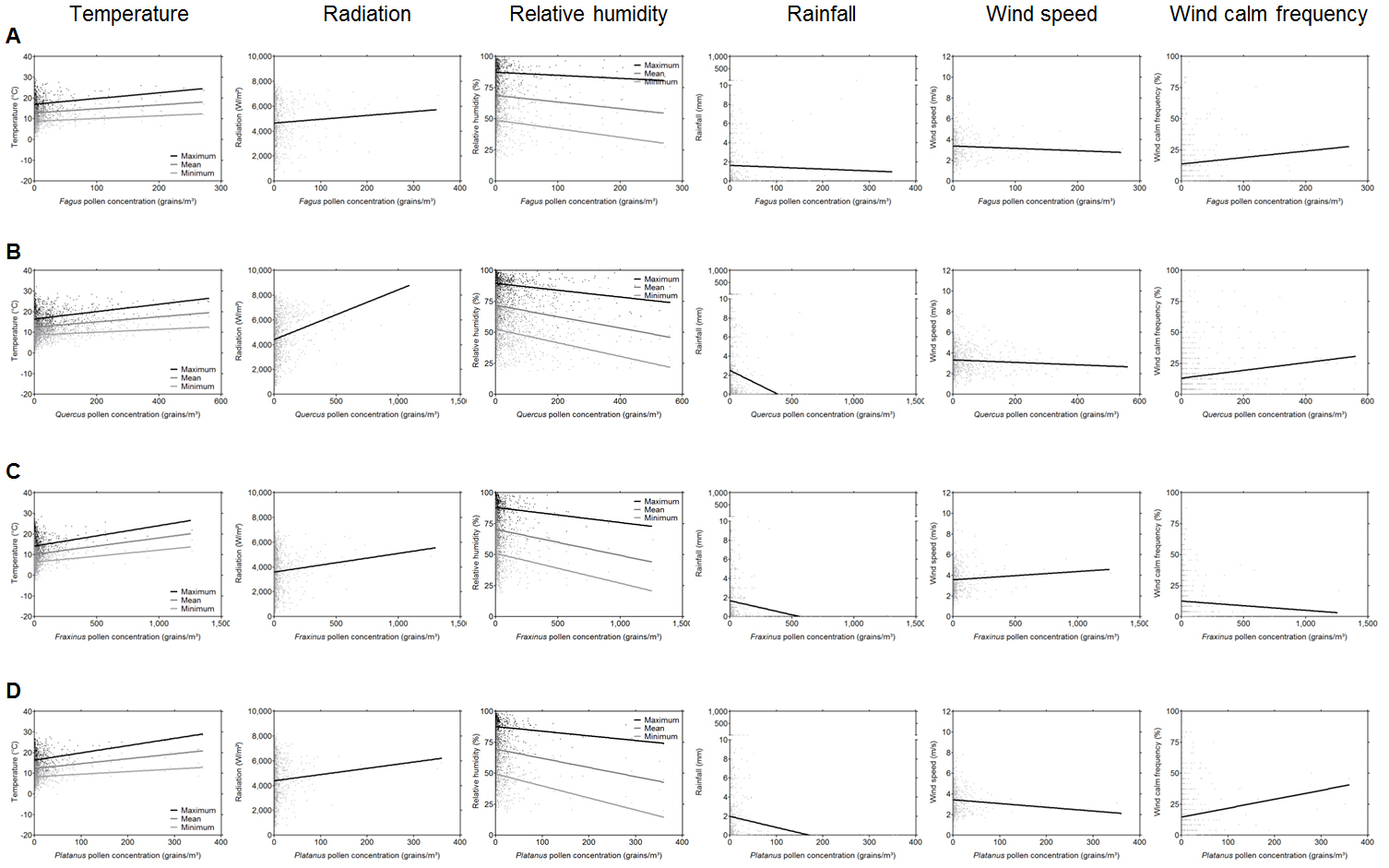

Supplement: Supplementary file 2 — Scatter plots of daily pollen concentrations for 4 tree taxa (A: beech, B: oak, C: ash, D: plane) vs. daily measures of 10 meteorological parameters. Datasets include measures between January 1st and September 30th, from 1982 to 2015. Trend was represented by linear regression. [file 484_2017_1457_MOESM2_ESM.jpg]

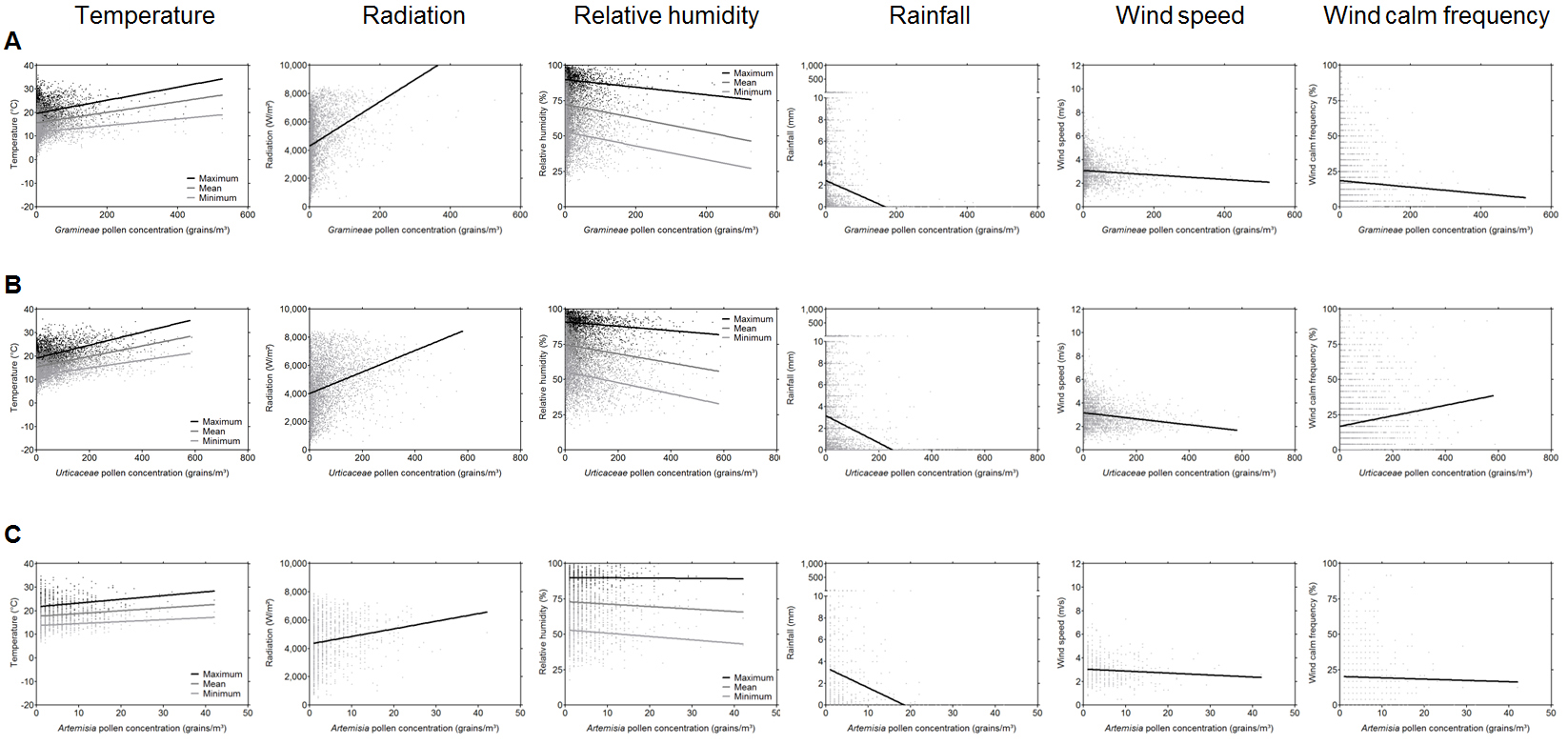

Supplement: Supplementary file 3 — Scatter plots of daily pollen concentrations for 3 herbaceous plant taxa (A: grasses, B: Urticaceae, C: mugwort) vs. daily measures of 10 meteorological parameters. Datasets include measures between January 1st and September 30th , from 1982 to 2015. Trend was represented by linear regression. [file 484_2017_1457_MOESM3_ESM.jpg]
